# Editorial: NAR Cancer and epigenetics and cancer

**DOI:** 10.1093/narcan/zcac003

**Published:** 2022-03-04

**Authors:** Danzhou Yang, Jonathan Dickerhoff, William S Dynan

**Affiliations:** Purdue University, College of Pharmacy, Medicinal Chemistry and Molecular Pharmacology, 575 W Stadium Ave., West Lafayette, IN 47907, USA; Purdue University Center for Cancer Research, 201 S University St, West Lafayette, IN 47906, USA; Purdue University, Department of Chemistry, West Lafayette, IN, USA; Purdue Institute for Drug Discovery, West Lafayette, IN, USA; Purdue University, College of Pharmacy, Medicinal Chemistry and Molecular Pharmacology, 575 W Stadium Ave., West Lafayette, IN 47907, USA; Emory University School of Medicine, Department of Radiation Oncology, Department of Biochemistry, and Winship Cancer Institute, 1510 Clifton Rd NE, Atlanta GA 30322, USA

Over the past 20 years, breakthrough discoveries in epigenetics have transformed our knowledge of chromatin structure alterations and regulation mechanisms in response to physiological or pathological signals. Without changing the gene sequence, epigenetic changes affect the way our genes work, leading to heritable phenotypes. Cancer epigenetics is one of the most active areas of cancer research and represents the single largest category of article submissions to *NAR Cancer*.

*NAR Cancer* is pleased to publish a thematic collection of articles, ‘Cancer Epigenetics’ that encapsulates exciting discoveries in this area. Ten Surveys and Summaries provide insights into the ways in which cancer progression, diagnosis and therapy are interwoven with epigenetics. These review articles, together with relevant recent standard articles, are now made available as a special collection on the journal’s website. This is the journal’s first-ever thematic collection. We plan for these collections to become regular features in the future.

Chemical modification of DNA and histones is the best-known example of epigenetic control. Methyltransferases and other modifying enzymes are crucial players in epigenetic regulation. Misregulation of DNA methyltransferases can lead to aberrant gene expression in cancer, as discussed by Mensah *et al.* (1). Polycomb repressive complexes are histone modifying proteins that have cancer-specific roles and are potential therapeutic targets, as presented by Wang *et al.* (2).

In prostate cancer, the ACK1 and HOXB13 cell survival pathways are important epigenetic players in pathogenesis, with the former also involved in histone phosphorylation, as described by Kim *et al.* (3).

The phosphorylation and dephosphorylation of modifying proteins affects their activity and thereby the epigenetic state of cancer cells. Tinsley and Allen-Petersen summarize the central role of protein phosphatase 2A complexes and how these might be leveraged as therapeutic targets (4).

Oxidative stress can cause chemical modification of DNA G-quadruplexes, which represents another type of epigenetic alteration. Radical oxygen species create 8-oxoguanine residues, which in turn affect the formation of regulatory G-quadruplexes, as discussed by Fleming and Burrows (5).

The epitranscriptome also encompasses epigenetic RNA modifications, which can affect gene expression. Kumari *et al.* showcase how these are involved in breast cancer (6).

Characteristic patterns of epigenetic modifications play a key role in cancer onset, as discussed by Lelievre (7). Thus, epigenome analysis has diagnostic potential to identify individuals with the highest cancer risk for early intervention.

Epigenetics also opens new strategies for cancer therapy. Kretzmann *et al.* describe epigenetic editing, together with other non-traditional approaches, for breast cancer therapy (8). A better understanding of epigenetics has also led to the discovery of new mechanism of actions for established small-molecule drugs. For example, the widely used chemotherapeutic drug 5-fluorouracil has been identified as an extrinsic source of epigenetic RNA modification, as described by Chalabi-Dchar *et al.* (9).

RNA-based therapeutics provide another possible way to regulate gene expression and down-regulate specific proteins. Delivery of therapeutic RNAs has been a major concern. Abdelaal and Kasinski summarize how ligand-mediated delivery might overcome this challenge (10).
